# Functional network resilience to pathology in presymptomatic genetic frontotemporal dementia

**DOI:** 10.1016/j.neurobiolaging.2018.12.009

**Published:** 2019-05

**Authors:** Timothy Rittman, Robin Borchert, Simon Jones, John van Swieten, Barbara Borroni, Daniela Galimberti, Mario Masellis, Maria Carmela Tartaglia, Caroline Graff, Fabrizio Tagliavini, Giovanni B. Frisoni, Robert Laforce, Elizabeth Finger, Alexandre Mendonça, Sandro Sorbi, Jonathan D. Rohrer, James B. Rowe, The Genetic Frontotemporal Dementia Initiative (GENFI), Sónia Afonso, Maria Rosario Almeida, Sarah Anderl-Straub, Christin Andersson, Anna Antonell, Silvana Archetti, Andrea Arighi, Mircea Balasa, Myriam Barandiaran, Nuria Bargalló, Robart Bartha, Benjamin Bender, Luisa Benussi, Valentina Bessi, Giuliano Binetti, Sandra Black, Martina Bocchetta, Sergi Borrego-Ecija, Jose Bras, Rose Bruffaerts, Paola Caroppo, David Cash, Miguel Castelo-Branco, Rhian Convery, Thomas Cope, Maura Cosseddu, María de Arriba, Giuseppe Di Fede, Zigor Díaz, Katrina M. Dick, Diana Duro, Chiara Fenoglio, Camilla Ferrari, Catarina B. Ferreira, Toby Flanagana, Nick Fox, Morris Freedman, Giorgio Fumagalli, Alazne Gabilondo, Roberto Gasparotti, Serge Gauthier, Stefano Gazzina, Roberta Ghidoni, Giorgio Giaccone, Ana Gorostidi, Caroline Greaves, Rita Guerreiro, Carolin Heller, Tobias Hoegen, Begoña Indakoetxea, Vesna Jelic, Lize Jiskoot, Hans-Otto Karnath, Ron Keren, Maria João Leitão, Albert Lladó, Gemma Lombardi, Sandra Loosli, Carolina Maruta, Simon Mead, Lieke Meeter, Gabriel Miltenberger, Rick van Minkelen, Sara Mitchell, Benedetta Nacmias, Mollie Neason, Jennifer Nicholas, Linn Öijerstedt, Jaume Olives, Alessandro Padovani, Jessica Panman, Janne Papma, Michela Pievani, Yolande Pijnenburg, Enrico Premi, Sara Prioni, Catharina Prix, Rosa Rademakers, Veronica Redaelli, Ekaterina Rogaeva, Pedro Rosa-Neto, Giacomina Rossi, Martin Rosser, Beatriz Santiago, Elio Scarpini, Sonja Schönecker, Elisa Semler, Rachelle Shafei, Christen Shoesmith, Miguel Tábuas-Pereira, Mikel Tainta, Ricardo Taipa, David Tang-Wai, David L. Thomas, Hakan Thonberg, Carolyn Timberlake, Pietro Tiraboschi, Philip Vandamme, Mathieu Vandenbulcke, Michele Veldsman, Ana Verdelho, Jorge Villanua, Jason Warren, Carlo Wilke, Ione Woollacott, Elisabeth Wlasich, Henrik Zetterberg, Miren Zulaica

**Affiliations:** t Instituto Ciencias Nucleares Aplicadas a Saude, Universidade de Coimbra, Coimbra, Portugal; u Faculty of Medicine, University of Coimbra, Coimbra, Portugal; v Department of Neurology, University of Ulm, Ulm, Germany; w Department of Clinical Neuroscience, Karolinska Institutet, Stockholm, Sweden; x Alzheimer’s disease and Other Cognitive Disorders Unit, Neurology Service, Hospital Clínic, Barcelona, Spain; y Biotechnology Laboratory, Department of Diagnostics, Spedali Civili Hospital, Brescia, Italy; z Fondazione IRCCS Ca’ Granda Ospedale Maggiore Policlinico, Neurodegenerative Diseases Unit, Milan, Italy; aa University of Milan, Centro Dino Ferrari, Milan, Italy; bb Cognitive Disorders Unit, Department of Neurology, Donostia University Hospital, San Sebastian, Gipuzkoa, Spain; cc Neuroscience Area, Biodonostia Health Research Institute, San Sebastian, Gipuzkoa, Spain; dd Imaging Diagnostic Center, Hospital Clínic, Barcelona, Spain; ee Department of Medical Biophysics, The University of Western Ontario, London, Ontario, Canada; ff Centre for Functional and Metabolic Mapping, Robarts Research Institute, The University of Western Ontario, London, Ontario, Canada; gg Department of Diagnostic and Interventional Neuroradiology, University of Tübingen, Tübingen, Germany; hh Istituto di Ricovero e Cura a Carattere Scientifico Istituto Centro San Giovanni di Dio Fatebenefratelli, Brescia, Italy; ii Department of Neuroscience, Psychology, Drug Research, and Child Health, University of Florence, Florence, Italy; jj Sunnybrook Health Sciences Centre, Sunnybrook Research Institute, University of Toronto, Toronto, Canada; kk Dementia Research Centre, Department of Neurodegenerative Disease, UCL Institute of Neurology, Queen Square, London, UK; ll Dementia Research Institute, Department of Neurodegenerative Disease, UCL Institute of Neurology, Queen Square, London, UK; mm Laboratory for Cognitive Neurology, Department of Neurosciences, KU Leuven, Leuven, Belgium; nn Fondazione IRCCS Istituto Neurologico Carlo Besta, Milano, Italy; oo Department of Clinical Neuroscience, University of Cambridge, Cambridge, UK; pp Centre for Neurodegenerative Disorders, Neurology Unit, Department of Clinical and Experimental Sciences, University of Brescia, Brescia, Italy; qq CITA Alzheimer, San Sebastian, Gipuzkoa, Spain; rr Laboratory of Neurosciences, Institute of Molecular Medicine, Faculty of Medicine, University of Lisbon, Lisbon, Portugal; ss Faculty of Biology, Medicine and Health, Division of Neuroscience and Experimental Psychology, University of Manchester, Manchester, UK; tt Baycrest Health Sciences, Rotman Research Institute, University of Toronto, Toronto, Canada; uu Department of Neurosciences, Psychology, Drug Research and Child Health (NEUROFARBA), University of Florence, Florence, Italy; vv Neuroradiology Unit, University of Brescia, Brescia, Italy; ww Alzheimer Disease Research Unit, McGill Centre for Studies in Aging, Department of Neurology & Neurosurgery, McGill University, Montreal, Québec, Canada; xx Neurologische Klinik, Ludwig-Maximilians-Universität München, Munich, Germany; yy Division of Clinical Geriatrics, Karolinska Institutet, Stockholm, Sweden; zz Department of Neurology, Erasmus Medical Center, Rotterdam, Netherlands; aaa Division of Neuropsychology, Hertie-Institute for Clinical Brain Research and Center of Neurology, University of Tübingen, Tübingen, Germany; bbb The University Health Network, Toronto Rehabilitation Institute, Toronto, Canada; ccc Centre of Neurosciences and Cell Biology, Universidade de Coimbra, Coimbra, Portugal; ddd Department of Neuroscience, Psychology, Drug Research and Child Health, University of Florence, Florence, Italy; eee Laboratory of Language Research, Centro de Estudos Egas Moniz, Faculty of Medicine, University of Lisbon, Lisbon, Portugal; fff MRC Prion Unit, Department of Neurodegenerative Disease, UCL Institute of Neurology, Queen Square, London, UK; ggg Faculty of Medicine, University of Lisbon, Lisbon, Portugal; hhh Department of Clinical Genetics, Erasmus Medical Center, Rotterdam, the Netherlands; iii Department of Medical Statistics, London School of Hygiene and Tropical Medicine, London, UK; jjj Department of Geriatric Medicine, Karolinska University Hospital-Huddinge, Stockholm, Sweden; kkk Amsterdam University Medical Centre, Amsterdam VUmc, Amsterdam, the Netherlands; lll London Ontario geneticist, Department of Neurosciences, Mayo Clinic, Jacksonville, FL, USA; mmm Tanz Centre for Research in Neurodegenerative Diseases, University of Toronto, Toronto, Canada; nnn Translational Neuroimaging Laboratory, McGill Centre for Studies in Aging, McGill University, Montreal, Québec, Canada; ooo Neurology Department, Centro Hospitalar e Universitario de Coimbra, Coimbra, Portugal; ppp Department of Clinical Neurological Sciences, University of Western Ontario, London, Ontario, Canada; qqq Neuropathology Unit and Department of Neurology, Centro Hospitalar do Porto - Hospital de Santo António, Oporto, Portugal; rrr The University Health Network, Krembil Research Institute, Toronto, Canada; sss Neuroimaging Analysis Centre, Department of Brain Repair and Rehabilitation, UCL Institute of Neurology, Queen Square, London, UK; ttt Center for Alzheimer Research, Division of Neurogeriatrics, Karolinska Institutet, Stockholm, Sweden; uuu Department of Clinical Neurosciences, University of Cambridge, Cambridge, UK; vvv Neurology Service, University Hospitals Leuven, Belgium; www Laboratory for Neurobiology, VIB-KU Leuven Centre for Brain Research, Leuven, Belgium; xxx Geriatric Psychiatry Service, University Hospitals Leuven, Belgium; yyy Neuropsychiatry, Department of Neurosciences, KU Leuven, Leuven, Belgium; zzz Nuffield Department of Clinical Neurosciences, Medical Sciences Division, University of Oxford, Oxford, UK; aaaa Department of Neurosciences and Mental Health, Centro Hospitalar Lisboa Norte - Hospital de Santa Maria & Faculty of Medicine, University of Lisbon, Lisbon, Portugal; bbbb OSATEK, University of Donostia, San Sebastian, Gipuzkoa, Spain; cccc Department of Neurodegenerative Diseases, Hertie-Institute for Clinical Brain Research and Center of Neurology, University of Tübingen, Tübingen, Germany; dddd Center for Neurodegenerative Diseases (DZNE), Tübingen, Germany; a Department of Clinical Neurosciences, University of Cambridge, Cambridge, UK; b Alzheimercentrum, Erasmus Medical Center, Rotterdam, the Netherlands; c Department of Clinical and Experimental Sciences, University of Brescia, Italy; d Department of Pathophysiology and Transplantation, “Dino Ferrari” Center, University of Milan, Fondazione Cà Granda, IRCCS Ospedale Maggiore Policlinico, Milan, Italy; e Cognitive Neurology Research Unit, Sunnybrook Health Sciences Centre, Toronto, Canada; f Hurvitz Brain Sciences Research Program, Sunnybrook Research Institute, Toronto, Canada; g Department of Medicine, University of Toronto, Toronto, Canada; h Tanz Centre for Research in Neurodegenerative Diseases, University of Toronto, Toronto, Canada; i Department NVS, Center for Alzheimer Research, Division of Neurogeriatrics, Karolinska Institutet, Stockholm, Sweden; j Department of Geriatric Medicine, Karolinska University Hospital, Stockholm, Sweden; k Istituto Neurologico Carlo Besta, Milan, Italy; l Department of Psychiatry, University Hospitals and University of Geneva, Geneva, Switzerland; m Neuroimaging and Epidemiology Unit, IRCCS San Giovanni di Dio Fatebenefratelli Brescia, Brescia, Italy; n Faculty of Medicine, Université Laval, Quebec, Canada; o Department of Clinical Neurological Sciences, University of Western Ontario, Ontario, Canada; p Faculdade de Medicina, Universidade de Lisboa, Lisboa, Portugal; q Department of Neurosciences, Psychology, Drug Research and Child Health (NEUROFARBA), University of Florence, Florence, Italy; r IRCCS Don Gnocchi, Florence, Italy; s Dementia Research Centre, Department of Neurodegenerative Disease, UCL Institute of Neurology, Queen Square, London, UK

**Keywords:** Frontotemporal dementia, Genetics, Connectivity, Functional imaging, Cognition

## Abstract

The presymptomatic phase of neurodegenerative diseases are characterized by structural brain changes without significant clinical features. We set out to investigate the contribution of functional network resilience to preserved cognition in presymptomatic genetic frontotemporal dementia. We studied 172 people from families carrying genetic abnormalities in C9orf72, MAPT, or PGRN. Networks were extracted from functional MRI data and assessed using graph theoretical analysis. We found that despite loss of both brain volume and functional connections, there is maintenance of an efficient topological organization of the brain's functional network in the years leading up to the estimated age of frontotemporal dementia symptom onset. After this point, functional network efficiency declines markedly. Reduction in connectedness was most marked in highly connected hub regions. Measures of topological efficiency of the brain's functional network and organization predicted cognitive dysfunction in domains related to symptomatic frontotemporal dementia and connectivity correlated with brain volume loss in frontotemporal dementia. We propose that maintaining the efficient organization of the brain's functional network supports cognitive health even as atrophy and connectivity decline presymptomatically.

## Highlights

• Mechanisms of preserved function in presymptomatic dementia are not well understood.
• We studied people with genetic frontotemporal dementia and their relatives.
• Brain network efficiency was preserved prior to the onset of symptoms.
• Highly connected hub regions were preferentially affected by neuropathology.
• Interventions to support functional brain networks may delay the onset of dementia.

## 1 Introduction

Many neurodegenerative dementias begin their neuropathology years or even decades before the onset of symptoms. The evidence of presymptomatic pathology comes from changes in structural brain imaging, positron emission tomograph ligands that bind to pathological proteins, and abnormal cerebrospinal fluid and blood biomarkers (Jack Jr et al., 2010, Ridha et al., 2006, Rohrer et al., 2015). However, it is not clear why people with significant progressive neurodegeneration and brain volume loss remain free of symptoms for so long or develop symptoms when they do. To address this issue, we assessed functional network resilience in the Genetic Frontotemporal Dementia Initiative (GENFI) cohort.

Network resilience derives from the robust and efficient arrangement of connections between brain regions (Bullmore and Sporns, 2012). This arrangement is characterized by the presence of highly connected hubs (Power et al., 2013, Tomasi and Volkow, 2011) in a “small world” arrangement which minimizes the topological distance (also called path length) between parts of the network. This path length can be used to derive measures of global or regional network efficiency. Networks that have an efficient small-world topology are intrinsically robust to processes that damage the network by removing network nodes or connections.

Examining the network organization of the brain has provided critical insights into neurocognitive development and diverse disorders of the nervous system from multiple sclerosis (Hawellek et al., 2011, Rocca et al., 2014), depression (Greicius et al., 2007), schizophrenia (Fornito et al., 2012), and autism (Moseley et al., 2015) to multiple neurodegenerative diseases including frontotemporal dementia (FTD) (Filippi et al., 2013, Seeley et al., 2007, Zhou et al., 2010); Alzheimer's disease, Parkinson's disease (Luo et al., 2014, Rittman et al., 2016); and Progressive Supranuclear Palsy (Rittman et al., 2016, Whitwell et al., 2011). In patients, altered network connectivity is consistently associated with the loss of cognitive function (Day et al., 2013, Pievani et al., 2011) or reduced response to treatment (Lui et al., 2011, Ye et al., 2016). In contrast, here we assess whether network integration provides resilience at earlier stages of the disease process, with the maintenance of cognitive well-being, even in the presence of established neuropathology and brain atrophy. To be more specific, we assess functional network resilience, which is defined as the maintenance of the topological properties of a functional brain network in the context of structural changes to the brain.

We identified functional brain networks from functional MRI (fMRI) images, using the blood oxygen level–dependent effect as an indirect measure of neural activity. The advent of task-free fMRI (also called “resting state” fMRI) (Biswal et al., 1997) has facilitated the analysis of brain function in severely impaired clinical groups while retaining a strong relationship to functionally defined brain networks. The connectome (Sporns, 2011) derived from task-free fMRI is robust, reproducible, and capable of generating brain networks analogous to other physiological techniques such as EEG or magnetoencephalography (Brookes et al., 2011).

We used task-free fMRI to assess people with genetic FTD and their first-degree relatives in whom approximately half carry the familial gene abnormality. Our cohort included mutations or expansions in the three major genes associated with FTD: PGRN, MAPT, C9orf72. We tested the hypothesis that before the age of symptom onset in genetic FTD, functional network resilience arises from the maintenance of an efficient network topology preserving cognitive function in the context of progressive pathology assessed by brain volume loss. From the age of symptom onset, we would expect the loss of functional network resilience, with a decline in network efficiency and connectivity in relation to both brain volume loss and cognitive function.

## 2 Materials and methods

Subjects were recruited as part of the multicenter international GENFI and underwent a standardized assessment (Rohrer et al., 2015). The age of expected symptom onset was defined as the mean within each family, which is significantly correlated among affected relatives (Rohrer et al., 2015). Echo-Planar Imaging and Magnetization Prepared Rapid Gradient Echo (MPRAGE) were acquired at each center. Analogous imaging sequences were acquired at each GENFI study site accommodating different manufacturers and field strengths (1.5 T and 3T). Echo-planar images were acquired over at least 300 s with a median of 315 s (inter-quartile range 309–440) and had a median repetition time of 2200 ms (2200 ms–3000 ms), echo time of 30 ms, in-plane resolution of 2.75 × 2.75 mm (2.75–3.31 × 2.75–3.31), and slice thickness of 3.3 mm (3.0–3.3). MPRAGE images were obtained during the same acquisition.

Image preprocessing used MPRAGE images to generate a transformation to register images to Montreal Neurological Institute standard space via a study-specific template using Diffeomorphic Anatomical Registration Through Exponentiated Lie algebra implemented in SPM12 (www.fil.ion.ucl.ac.uk/spm/software/spm12/). This transformation was applied to coregistered functional images. Functional image pre-processing was performed using the brainwavelet pipeline (www.brainwavelet.org) including slice-time correction, regression of cerebrospinal fluid, white matter, movement parameters and their derivatives, and despiking using a wavelet algorithm. Identification of motion outliers for exclusion used the spike percentage threshold, defined as the percentage of the time series in which spikes were identified during the wavelet despiking process. The spike percentage threshold was set at 10% at which level the removal of subjects did not significantly change the connection strength measured across all subjects.

Each subject's brain volume was parcellated into 500 approximately equally sized regions using a centroidal Voronoi tessalation (Du et al., 1999). Of the 500 regions, 29 were insufficiently covered in some or all subjects, leaving 471 regions for further analysis. The fMRI signal time series within each parcel was bandpass filtered using a wavelet scale of 0.0675–0.125 Hz.

Graph theoretical analysis was applied to network connectivity; the wavelet cross-correlation was used as a measure of the strength of each connection. Networks were then analyzed in terms of connection strength, efficiency, and connectedness. Graph analysis used the Maybrain package (github.com/RittmanResearch/maybrain). We defined connection strength as the sum of nodal connection strength (also called weighted degree) values of all the network's nodes. To capture the property of network efficiency, we use measures based on path length. The global efficiency is defined as the sum of the inverse path lengths for all nodes in a network. The analogous nodal measure of closeness centrality is defined as the sum of the path lengths for each node to all other network nodes. Efficiency measures were normalized against the mean value generated from 500 graphs with an identical degree distribution and random connections. We assessed atrophy by calculating the percentage brain volume or regional volume compared with the total intracranial volume. Hubs were defined in the gene-negative group as brain regions with connection strength two standard deviations greater than other regions.

Because network measures are not independent, we did not apply correction for multiple comparisons. Group comparisons between the gene carrier and FTD group were performed for each network measure using a mixed-effects linear model with diagnostic group as the main effect, age as a dependent variable, and scan site and gene type as random variables using the lmer package in R. We included the gene-negative group in all models to properly estimate the effect of age. We then assessed group differences by specifying an appropriate contrast between the gene carrier group and FTD groups. The Satterthwaite estimate of effective degrees of freedom enabled calculation of significance values. To assess the relationship between estimated age at onset and network measures, we extended the linear mixed-effects model by including an interaction term between the diagnostic group and estimated time to symptom onset.

## 3 Results

Twenty-nine people with genetic FTD were recruited (12 C9orf72, 11 MAPT, 6 PGRN), 70 unaffected relatives carrying the same mutation we will refer to as “gene carriers” (17 C9orf72, 13 MAPT, 40 PGRN) and 86 relatives without the mutation referred to as “gene negative.” During image processing, 13 subjects were removed because of excessive motion, 5 with FTD (1 C9orf72, 2 MAPT, 2 PGRN), 2 gene carriers (2 PGRN), and 6 gene negative. The remaining 172 subjects were taken forward for analysis: 24 FTD, 68 gene carriers, and 80 gene negative. Demographic information is shown in Table 1. The FTD clinical syndromes were behavioral variant FTD n = 20, FTD-motor neuron disease n = 1, primary progressive aphasia n = 2, dementia not otherwise specified = 1.

**Table 1:** Demographics for subjects included in the analysis

Demographic	*p* Value	Gene negative	Gene carriers	FTD
Age, y (SD)	<0.00001	47.8 (15.5)	44.5 (12.3)	62.4 (8.6)
Sex (M/F)	ns^a^	49(61%)/31(39%)	40(59%)/28(41%)	7(29%)/17(71%)
Hand (L/R/Ambi)	ns	74(93%)/5(6%)/1 (1%)	58(85%)/8(12%)/2 (3%)	22(92%)/2(8%)/0 (0%)
Education, y (SD)	ns	13.7 (3.5)	13.8 (3.2)	12.2 (4.5)

For parametric data, analysis of variance was used and we report the mean, and the standard deviation in parentheses.

For categorical data, the χ^2^ test was used and we report the numbers in each category.

As expected, people with FTD were older than both gene carriers (*p* < 0.00001) and gene-negative subjects (*p* < 0.00001).

Key: FTD, frontotemporal dementia; ns, non-significant >0.1; SD, standard deviation.

a Although sex differences were not significant when tested across all three groups, pairwise tests confirmed that there were fewer men in the FTD patient group compared with both the gene carrier (*p* = 0.02) and gene-negative (*p* = 0.01) groups.

### 3.1 Differences in network connectivity and efficiency between groups

**Fig. 1:**
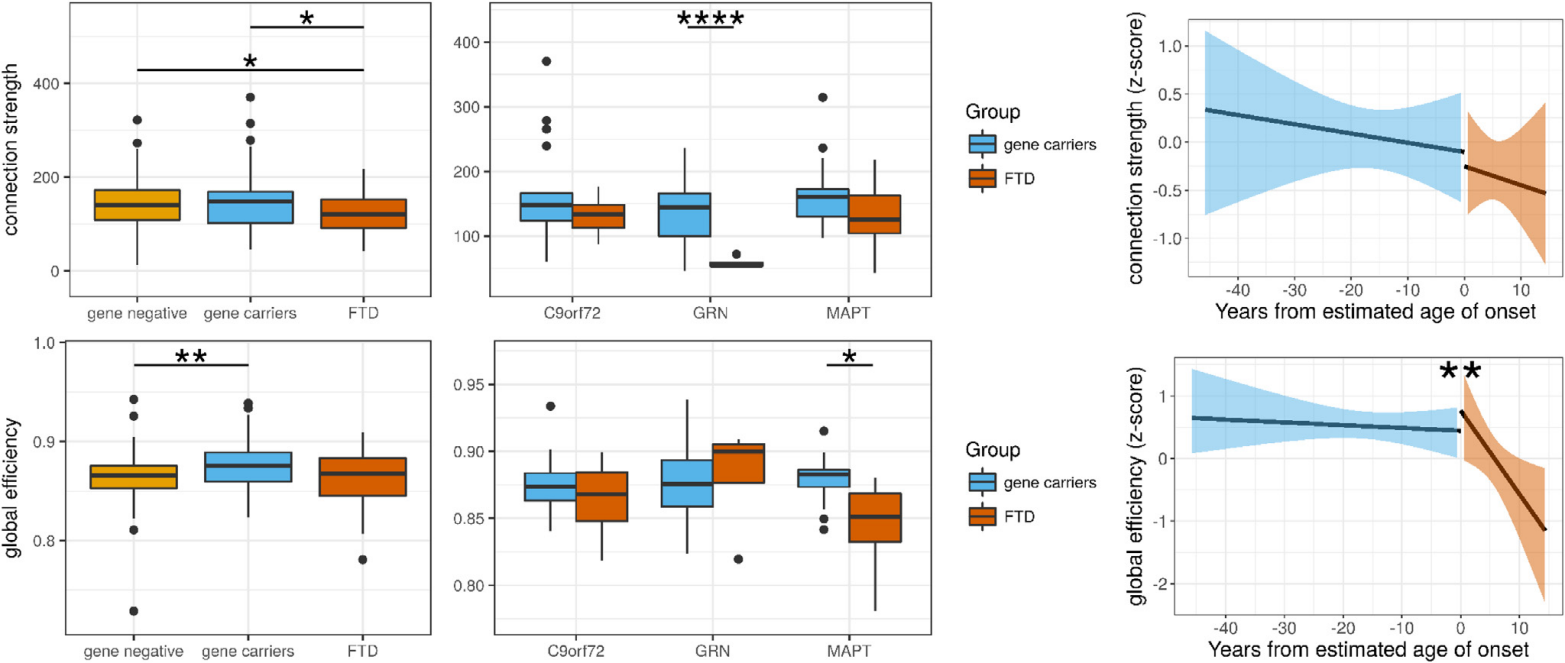
Connection strength is reduced in genetic FTD compared to asymptomatic gene–carrying relatives. Differences between the genetic FTD group and presymptomatic-gene carrying relatives demonstrate reduced connection strength using a mixed-effects linear model (*p* = 0.01) with no difference in global efficiency (*p* = 0.2). The results for individual genes are shown for completeness, although we would be cautious in interpreting these results, given the small group sizes. Using a simple *t*-test, there was significantly reduced connection strength in the PGRN FTD group (*p* < 0.00001) and global efficiency in the MAPT FTD group (*p* = 0.02). To assess whether there was a nonlinear relationship between network measures and time to the estimated age of symptom onset, we performed discontinuous breakpoint analysis. There was a significant breakpoint in global efficiency (*p* = 0.009) but not for connection strength (*p* = 0.9). Significance values: ^∗^<0.05, ^∗∗^<0.01, ^∗∗∗^<0.001. FTD, frontotemporal dementia.

**Fig. 2:**
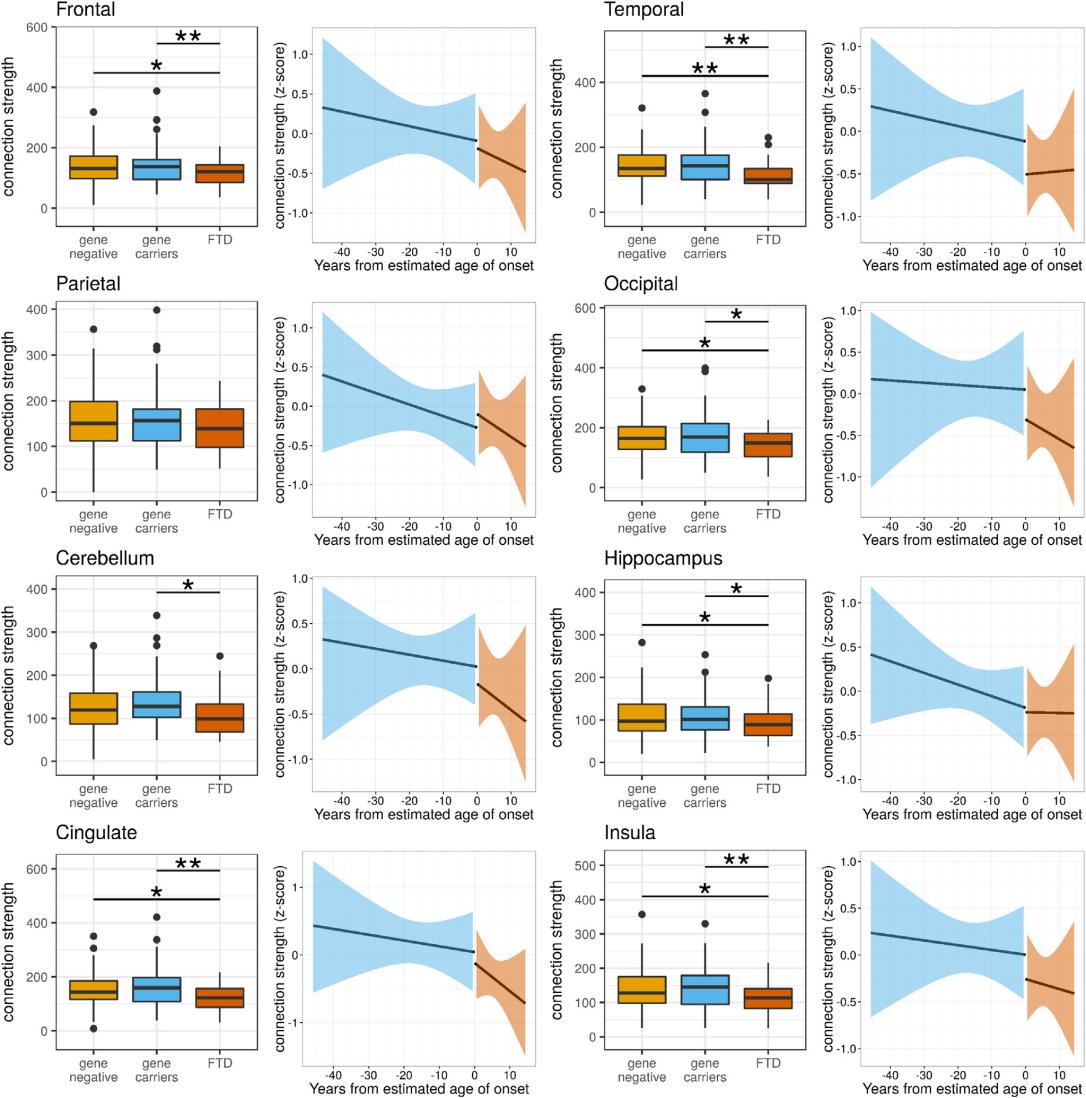
Although relevant brain regions demonstrate reduced connectivity in FTD, there is no significant change at symptom onset. For each brain region, the difference in connection strength between gene carrier and FTD groups is presented; significant values were calculated using a mixed-effects linear regression model. There were significant differences in the frontal, temporal, occipital, cingulate, and insula cortices (see eResults). However, no brain region demonstrated a significant breakpoint in connect strength at the age of symptom onset (using a piecewise linear regression model). Significance values: ^∗^<0.05, ^∗∗^<0.01, ^∗∗∗^<0.001. FTD, frontotemporal dementia.

**Fig. 3:**
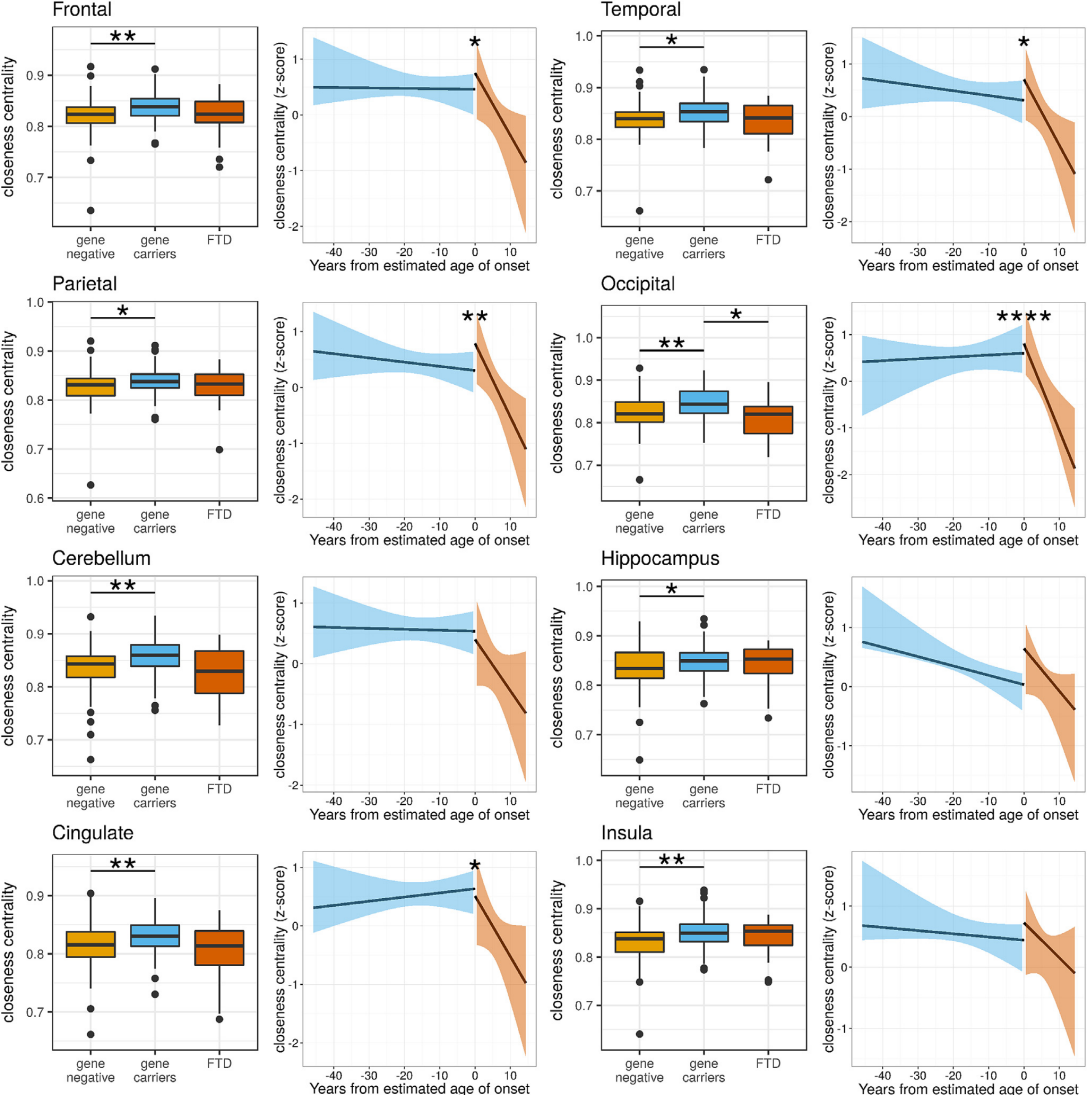
Brain regions demonstrate both reduced efficiency in FTD and a significant decline in efficiency beginning at symptom onset. For each brain region, the difference in closeness centrality between gene carrier and FTD groups are presented; significant values were calculated using a mixed-effects linear regression model (see eResults). There were significant differences in the frontal, temporal, occipital, cerebellar, and cingulate cortices. In contrast to the connectivity results, there were significant breakpoints in closeness centrality at the age of symptom onset in frontal, temporal, parietal, occipital, and cingulate cortices. These findings suggest that an efficient brain structure is maintained in these brain regions up to the time that symptoms of FTD emerge but that the efficient structure rapidly breaks down thereafter. Significance values: ^∗^<0.05, ^∗∗^ <0.01. FTD, frontotemporal dementia.

To assess the difference in global network properties between the gene negative, gene carriers, and FTD groups, brain networks were assessed for connection strength and global efficiency, shown in Fig. 1. The FTD group (mean connection strength 121.8) was less well connected compared with gene carrier (149.4, *p* = 0.01) and gene-negative groups (147.1, *p* = 0.02). Gene carriers (mean global efficiency 0.88) had a higher global efficiency than the gene-negative group (0.86, *p* = 0.004), but there was no differences in global efficiency in any other comparison (FTD 0.86). We found similar regional reduction in connectivity in frontal lobes, temporal lobes, occipital lobes, and cingulate cortices, cerebellum, and insula cortices in the FTD group compared with gene carriers; increased efficiency (closeness centrality) in all brain regions in the gene carrier group compared with the gene-negative group and reduced efficiency in the occipital cortex in FTD compared with gene carriers; see Fig. 2, Fig. 3 and eTable 1.

To assess whether regional network properties would influence change in network properties, we examined the most highly connected “hub” regions. By definition, hubs were more connected than nonhubs; however, the difference in connection strength between hubs and nonhubs was significantly smaller in the FTD group (*p* = 0.02), suggesting that hubs were weaker in the FTD group. The difference in efficiency measured by closeness centrality between hubs and nonhubs was abolished in the FTD group (effect size 0.0025, *p* = 0.5) compared with gene carriers (effect size −0.01, *p* < 0.00001), the difference between these effects being significant (*p* = 0.001).

### 3.2 Disease progression and network measures

To test the relationship between network measures and disease progression, we began by estimating the temporal relationships between network measures and symptom onset. There were no simple linear relationships of time to the estimated age of symptom onset with connection strength (*p* = 0.6) or global efficiency (*p* = 0.17).

We then tested whether there may be a nonlinear decline in network properties. We assessed whether a breakpoint existed in the relationship between estimated time to symptom onset and network measures at the estimated time of symptom onset using piecewise regression analysis. There was no significant breakpoint in network measures at the estimated time of onset in connection strength for the whole brain (*p* = 0.9) or any brain region, see Fig. 2 and eResults. For global efficiency, we found a significant breakpoint (*p* = 0.009) suggesting that global efficiency starts to decline at the time of symptom onset, see Fig. 1. We saw similar breakpoints for efficiency in the frontal lobes, parietal lobes, occipital lobes, and cingulate cortex, see Fig. 3 and eResults. These results suggest that network topology declines in a dramatic fashion at the point of transition from presympomatic to symptomatic FTD.

### 3.3 Functional network resilience to brain atrophy

**Fig. 4:**
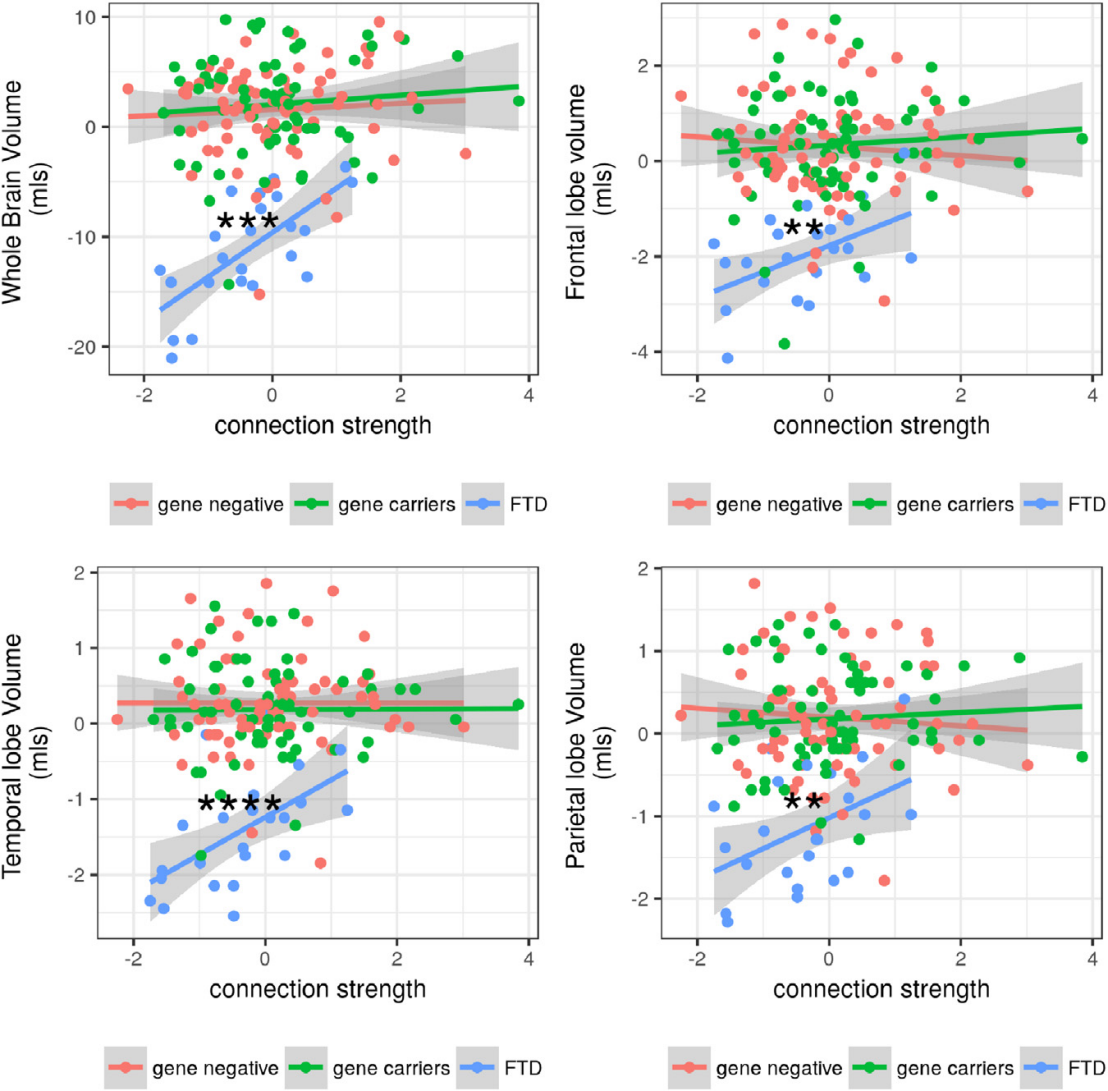
Whole-brain atrophy and the atrophy in relevant brain regions are correlated with the loss of connectivity only after symptom onset. We examined whether the volume of the whole brain and brain regions was associated with loss of connection strength. There was a relationship between volume and connection strength in the whole brain (*p* = 0.0002), frontal lobe (*p* = 0.005), and temporal lobes (*p* < 0.00001) in the FTD group only and not in the gene carrier group; in each case, there was a significant difference between the relationship in the FTD group and gene carrier groups (whole-brain *p* = 0.001; frontal lobes *p* = 0.02; temporal lobes *p* = 0.0002). Significance values: ^∗∗^<0.01, ^∗∗∗^<0.001, ^∗∗∗∗^<0.0001. FTD, frontotemporal dementia.

We assessed whether connection strength and network efficiency was associated with brain volume loss, see Fig. 4. Connection strength correlated with reduced brain volume in the FTD group (r = 0.47, *p* = 0.0002). This correlation differed significantly from the nonsignificant relationship between connection strength in the gene carrier group (r = 0.031, *p* = 0.6), difference between interactions (*p* = 0.001). Similar differences were seen in the frontal, temporal, and parietal lobes; see Fig. 4 and eResults.

There was no relationship between global efficiency in the FTD group and whole-brain atrophy (*p* = 0.2), and no interaction between the FTD group and gene carriers on the relationship between global efficiency and whole-brain atrophy (*p* = 0.3). No brain regions demonstrated a relationship between global efficiency and whole-brain or regional atrophy.

### 3.4 Relationship between network properties and cognitive function

Clinical scores are shown in Table 2. As expected, there were no significant differences between gene-negative and gene carrier groups, whereas all measures were markedly impaired in the FTD group compared with the gene carrier group (*p* < 0.0001 for all comparisons). The relationship between clinical test scores and years from expected onset was not clearly linear in the FTD group, suggestive of an acute decline in ability at diagnosis rather than a continuous linear association.

**Table 2:** Mean clinical scores for each group with standard deviation shown in parentheses

	Gene negative	Gene carriers	FTD
MMSE	29.2 (1.4)	29.1 (1.5)	22.3 (6.3)
Log immediate memory	0.08 (1.02)	0.08 (0.84)	−2.07 (1.1)
Log delayed memory	0.08 (0.98)	−0.04 (0.77)	−2.08 (0.99)
Forward digit span	0.02 (0.97)	−0.03 (1)	−1.21 (1.44)
Backwards digit span	0.01 (0.99)	−0.12 (0.9)	−1.71 (1.19)
Trails A	0.2 (0.91)	0.29 (0.58)	−2.49 (2.49)
Trails B	0.16 (0.91)	0.24 (0.88)	−2.49 (1.34)
Digit symbol task	0.25 (1.12)	0.27 (0.95)	−1.98 (0.89)
Boston Naming Task	0.15 (0.88)	0.03 (1.1)	−3.53 (2.66)
Verbal fluency (Category)	0.14 (1.02)	0.16 (0.91)	−2.04 (0.9)
Verbal fluency (Letter)	−0.06 (1.01)	−0.05 (1.2)	−2.64 (0.96)
Block design task	0.01 (1)	0.17 (0.98)	−2.05 (0.97)

The raw MMSE score is shown and z-score for other measures.

These scores are corrected for language but not for other demographics.

Key: FTD, frontotemporal dementia.

We found strong relationships in the FTD group of connection strength with both Mini-Mental State Examination (MMSE; *p* = 0.002) and trails A (*p* = 0.0002) and a difference in the relationships between the FTD and gene carrier groups for both cognitive measures (MMSE: *p* = 0.004, trails A: *p* = 0.0006), although there were possible ceiling effects in the gene carrier group on both these tests, see eTable 2 for full results.

For digit span and verbal fluency, we observed a relationship between connection strength and test performance across both FTD and gene carrier group combined, but no difference in the relationship between groups: digit span (*p* = 0.03), categorical verbal fluency (*p* = 0.03), and letter verbal fluency (*p* = 0.01). This suggests that a loss of connectivity before the onset of clinical symptoms is relevant to declining cognitive performance in these tests. Of note, we included age as a covariate in these models, to reduce the likelihood that age explained these results.

Higher global efficiency was associated with better performance on the MMSE in the gene carrier group (*p* < 0.001), but there was no such relationship in the FTD group (*p* = 0.053); the difference in the effect between groups was significant (*p* = 0.049). There was a decline in performance on Trails B with reduced global efficiency in the FTD group (*p* = 0.02), although the difference in this relationship from the gene carrier group did not reach significance (*p* = 0.1). There was no other significant relationship between global efficiency and cognitive performance.

Finally, we tested whether region-specific measures might correlate with cognitive scores, shown in eTable 2. Both MMSE and Trials A demonstrated consistent relationships with connection strength in FTD and significant difference from the gene carrier group (occipital lobe, temporal lobe, insula, cingulate, hippocampus) similar to the whole-brain results. However, these tests demonstrate marked ceiling effects, which may limit the interpretation of these results.

Worse performance on forward digit span was related to a loss of connection strength in the parietal lobe in FTD and in the Boston naming test with loss of connection strength in the occipital lobe. Both these relationships differed significantly from the gene carrier group; see eTable 2.

For the network efficiency measure of closeness centrality, the Trials B test that requires significant working memory was related to network efficiency in the hippocampus, and this relationship differed significantly from the gene carrier group; see eTable 2. Similar to connection strength, there was a relationship between efficiency and MMSE score, and a significant difference in this relationship compared with the gene carrier group in the occipital lobe, cerebellum, and insula.

Taken together, the correlations with cognitive scores suggest that changes to specific brain regions of connection strength and efficiency may be relevant to specific cognitive functions, particularly in the Trails B, forward digit span, and Boston naming tasks.

## 4 Discussion

We demonstrate that the brain can function normally for cognitive well-being despite substantial presymptomatic neurodegenerative disease if it can maintain efficient information processing through functional connections, but that brain network efficiency declines sharply around the time of symptom onset. The loss of network efficiency is most severe in highly connected hub regions, and regional changes in network efficiency are associated with worsening of cognitive deficits associated with FTD. We propose that interventions during the crucial presymptomatic period of neurodegenerative disease could be effective if they promote the maintenance or resilience of the brain's intrinsically efficient arrangement of functional network connections.

Our findings challenge the concept that functional deficits mirror structural change early in the disease process. This is not to say that structural changes are irrelevant to brain function (Jack Jr et al., 2004, Jack Jr et al., 2009). However, many years before symptom onset, there can be gross changes in brain structure and cerebrospinal fluid biomarkers that indicate an active neuropathological processes and atrophy, both in familial neurodegenerative disease (Dopper et al., 2013, Ridha et al., 2006, Rohrer et al., 2015, Schott et al., 2003) and in sporadic disease such as early Alzheimer's disease and MCI (Liu et al., 2010, Olsson et al., 2016, Yao et al., 2010). We therefore tested whether resilience of brain network organization can explain the discrepancy between changes in structure and cognitive function.

The brain's resilience to structural change in presymptomatic disease might depend on topological resilience or active compensation. We propose that topological resilience provides a greater contribution for several reasons. In common with many ecological and man-made networks, the brain's network has a “small-world” configuration that balances the metabolic costs of long-distance connections between any two points in the network (path length) and shared connections between locally connected nodes (clustering) (Achard and Bullmore, 2007, Achard et al., 2006, Vértes et al., 2012). Highly connected hubs are essential to small-world networks. In the brain, they are metabolically active (Achard et al., 2012, Buckner et al., 2009) and play a role in efficient integration of information between regions (Power et al., 2013, Sepulcre et al., 2013, Sporns et al., 2007, Tomasi and Volkow, 2011). The presence of hubs means that small-world networks are resilient to targeted and random network attacks even if the hubs themselves are more prone to the effects of neuropathology.

The concept of functional network resilience is closely linked and overlapping with the concepts of cognitive reserve, brain reserve, and brain maintenance (Stern et al., 2018). Our definition of functional resilience is closely aligned with cognitive reserve, which is a multifaceted concept positing that educational, social, and exercise lead to maintained cognitive abilities in the context of aging or neurodegeneration (Cabeza et al., 2018). There is preliminary evidence that cognitive reserve (at least as estimated from academic and occupational attainments) ameliorates the cognitive impact of neurodegenerative disease or against reaching the threshold for diagnosis of neurodegenerative disease (Stern, 2009, Wu et al., 2016). Indeed, higher cognitive reserve (estimated by years of education) is associated with slower atrophy and later symptom onset in familial FTD associated with TAR DNA-binding protein 43 (Premi et al., 2017). This effect is moderated by genetic factors (TMEM106B genotype), with many questions remaining as to the mechanisms of effect of cognitive reserve. It is likely that functional brain imaging reflects aspects of cognitive reserve (Solé-Padullés et al., 2009), but these are not yet well established. It is beyond the scope of this study to identify the effect of education on functional network resilience or the genetic moderators of such an effect. As a cross-section study, possible cohort effects mean that differences in cognitive reserve between younger and older gene carriers cannot wholly be ruled out as a contributor to the maintenance of global efficiency we observe. However, the stability of global efficiency in the years leading up to symptom onset (Fig. 1) averages across subjects with differences in education and occupation reserve at any given range of years from expected onset of symptoms.

We found a complex relationship between functional connectivity and brain volume loss. In the FTD group, a relatively small reduction in connection strength was correlated with a much greater reduction in brain volume, which was not the case in presymptomatic or gene-negative participants. One intriguing possibility is that premorbid connection strength influences the rate of volume loss in disease. This echoes previous studies showing that specific brain network and connectivity patterns influence the pattern of brain atrophy and neuropathology in a range of neurodegenerative diseases (Cope et al., 2018, Seeley et al., 2009).

We assessed whether clinical measures of disease would help us to relate domains of cognitive function to the changes we observed in functional network resilience. In general, the associations were not strong, which may relate to the global nature of the network measures we assessed in comparison to the more specific and localizable clinical measures. However, we identified a decline in verbal fluency in relation to connection strength that may reflect subtle presymptomatic cognitive impairment. We found relationships between local measures of network connectivity with the Boston naming test in the occipital lobe and digit span in the parietal lobe. We are cautious about interpreting these results, given the relatively weak associations and the seeming mismatch in localization. It is likely that more local or network-specific measures of network integrity would be better associated with cognitive tests.

Our study has several important limitations. Cohorts of genetic dementia are rare and despite a coordinated multinational recruitment effort, the number of subjects is relatively small, although larger than many comparable studies of functional neuroimaging in dementia. This study was cross-sectional rather than longitudinal; therefore, our inference of change over time is based on the assumption of a similar starting value and rate of change between individuals. fMRI has been often open to criticism as a technique because it measures an indirect measure of blood oxygen level–dependent effect as a surrogate for neuronal activity (Tsvetanov et al., 2015); it has a poor frequency resolution, and it may be affected by movement of subjects within the scanner. Despite these limitations, it has proven to be a valuable and useful tool to interrogate brain networks and produces network data comparable to other techniques such as EEG or MEG. There were more females in the FTD group compared to males, although comparison across the three groups (gene negative, carriers, and FTD) was not significant. While a more balanced cohort would be ideal, we consider that the effects of FTD would outweigh any subtle gender effects, and gender differences would not explain the differences between gene carriers and gene-negative participants.

## 5 Conclusions

We propose that the maintenance of functional brain networks underlies the resilience of the brain to neurodegenerative pathology in the presence of significant neuronal loss. We suggest that resilient topological organization rather than active compensation is the main contributor to this resilience. Our findings suggest a window of opportunity to intervene in the presymptomatic stage of neurodegenerative diseases, including treatment strategies that promote efficiency and integration in the brain's functional brain networks even in the presence of progressive atrophy.

## Disclosure

The authors have no actual or potential conflicts of interest.
