# Supplementary material for: Functional network resilience to pathology in presymptomatic genetic frontotemporal dementia
Source: Neurobiol Aging. 2019 May;77:169–77. doi: 10.1016/j.neurobiolaging.2018.12.009 (PMC6491498; doi:10.1016/j.neurobiolaging.2018.12.009)
Supplement: Supplementary Material [file mmc1.doc]

# Supplementary material

Supplementary Table 1

|  | Connection strength | | | | | | Closeness centrality | | | | | |
| --- | --- | --- | --- | --- | --- | --- | --- | --- | --- | --- | --- | --- |
|  | FTD | | | FTD vs gene carriers | | | FTD | | | FTD vs gene carriers | | |
|  | t | df | p | t | df | p | t | df | p | t | df | p |
| **Frontal lobe functional network** | | |  |  |  |  |  |  |  |  |  |  |
| Whole brain | 3.90 | 89.2 | **0.0002** | -3.44 | 87.3 | **0.0009** | -1.11 | 90.6 | 0.3 |  |  |  |
| Frontal | 2.87 | 85.9 | **0.005** | -2.37 | 84.9 | **0.02** | -2.13 | 87.2 | **0.04** | 1.78 | 83.5 | 0.08 |
| Temporal | 4.27 | 89.1 | **<0.00001** | -3.85 | 88.0 | **0.0002** | -1.87 | 88.7 | 0.07 |  |  |  |
| Parietal | 3.24 | 86.4 | **0.002** | -2.82 | 85.3 | **0.006** | -2.50 | 90.8 | **0.01** | 0.88 | 88.1 | 0.4 |
| Occipital | 1.87 | 90.3 | 0.07 |  |  |  | 0.15 | 88.3 | 0.9 |  |  |  |
| Insula | 0.99 | 86.5 | 0.3 |  |  |  | 0.42 | 90.8 | 0.7 |  |  |  |
| Hippocampus | 1.27 | 90.3 | 0.2 |  |  |  | -0.49 | 88.9 | 0.6 |  |  |  |
| Cingulate | 2.24 | 90.8 | **0.03** | -1.67 | 90.7 | 0.1 | -1.93 | 79.5 | 0.06 |  |  |  |
| Amygdala | 0.59 | 88.3 | 0.6 |  |  |  | 0.49 | 88.7 | 0.6 |  |  |  |
| Caudate | 0.68 | 88.9 | 0.5 |  |  |  | -1.00 | 91.0 | 0.3 |  |  |  |
| Putamen | 1.01 | 90.8 | 0.3 |  |  |  | 1.48 | 89.6 | 0.1 |  |  |  |
| Thalamus | 1.78 | 90.5 | 0.08 |  |  |  | 0.99 | 89.6 | 0.3 |  |  |  |
|  |  |  |  |  |  |  |  |  |  |  |  |  |
| **Temporal lobe functional network** | | |  |  |  |  |  |  |  |  |  |  |
| Whole brain | 3.85 | 89.3 | **0.0002** | -3.34 | 87.1 | **0.001** | -1.09 | 90.8 | 0.3 |  |  |  |
| Frontal | 3.19 | 85.6 | **0.02** | -2.66 | 83.8 | **0.01** | -1.80 | 87.7 | 0.08 |  |  |  |
| Temporal | 4.27 | 89.5 | **<0.00001** | -3.97 | 87.2 | **0.0002** | -1.30 | 88.8 | 0.2 |  |  |  |
| Parietal | 3.51 | 86.6 | **0.0007** | -2.98 | 84.5 | **0.004** | -2.44 | 90.8 | **0.02** | 0.96 | 89.3 | 0.3 |
| Occipital | 2.11 | 90.2 | **0.04** | -1.89 | 87.6 | 0.06 | 0.36 | 88.1 | 0.7 |  |  |  |
| Insula | 1.46 | 87.0 | 0.2 |  |  |  | 1.45 | 90.8 | 0.2 |  |  |  |
| Hippocampus | 1.14 | 90.0 | 0.3 |  |  |  | -0.93 | 88.6 | 0.4 |  |  |  |
| Cingulate | 2.72 | 90.6 | **0.008** | -1.89 | 90.5 | 0.06 | -1.25 | 84.3 | 0.2 |  |  |  |
| Amygdala | 0.29 | 88.5 | 0.8 |  |  |  | 0.57 | 88.4 | 0.6 |  |  |  |
| Caudate | 1.00 | 89.6 | 0.3 |  |  |  | -0.18 | 91.0 | 0.9 |  |  |  |
| Putamen | 1.14 | 91.0 | 0.3 |  |  |  | 1.27 | 88.4 | 0.2 |  |  |  |
| Thalamus | 1.58 | 90.4 | 0.1 |  |  |  | 0.91 | 89.4 | 0.4 |  |  |  |
|  |  |  |  |  |  |  |  |  |  |  |  |  |
| **Parietal lobe functional network** | | | |  |  |  |  |  |  |  |  |  |
| Whole brain | 3.98 | 89.1 | **0.0001** | -3.56 | 86.7 | **0.0006** | -1.50 | 90.6 | 0.1 |  |  |  |
| Frontal | -3.15 | 13.4 | **0.008** | -2.56 | 83.9 | **0.01** | -2.07 | 90.0 | **0.04** | 1.74 | 84.8 | 0.09 |
| Temporal | 4.61 | 88.9 | **<0.00001** | -4.13 | 87.1 | **<0.00001** | -1.22 | 87.8 | 0.2 |  |  |  |
| Parietal | 2.99 | 86.3 | **0.004** | -2.62 | 84.5 | **0.01** | -3.03 | 90.5 | **0.003** | 1.32 | 88.7 | 0.2 |
| Occipital | 1.68 | 90.2 | 0.1 |  |  |  | 0.04 | 85.0 | 1.0 |  |  |  |
| Insula | 1.11 | 86.4 | 0.3 |  |  |  | 1.20 | 90.3 | 0.2 |  |  |  |
| Hippocampus | 1.14 | 90.3 | 0.3 |  |  |  | -0.58 | 89.5 | 0.6 |  |  |  |
| Cingulate | 2.11 | 91.0 | **0.04** | -1.46 | 89.9 | 0.2 | -1.89 | 57.2 | 0.06 |  |  |  |
| Amygdala | 0.54 | 88.6 | 0.6 |  |  |  | 0.96 | 89.0 | 0.3 |  |  |  |
| Caudate | 0.38 | 88.9 | 0.7 |  |  |  | 0.23 | 90.1 | 0.8 |  |  |  |
| Putamen | 0.77 | 90.7 | 0.4 |  |  |  | 0.98 | 81.8 | 0.3 |  |  |  |
| Thalamus | 1.44 | 90.3 | 0.2 |  |  |  | 0.37 | 90.6 | 0.7 |  |  |  |
|  |  |  |  |  |  |  |  |  |  |  |  |  |
| **Occipital lobe functional network** | | | |  |  |  |  |  |  |  |  |  |
| Whole brain | 3.62 | 89.7 | **0.0005** | -3.20 | 87.3 | **0.002** | -0.90 | 86.6 | 0.4 |  |  |  |
| Frontal | 2.85 | 85.6 | **0.006** | -2.47 | 83.7 | **0.02** | -1.71 | 87.7 | 0.09 |  |  |  |
| Temporal | 3.57 | 89.3 | **0.0006** | -3.38 | 87.3 | **0.001** | -1.46 | 78.2 | 0.2 |  |  |  |
| Parietal | 2.70 | 86.4 | **0.008** | -2.30 | 84.7 | **0.02** | -2.50 | 85.8 | **0.01** | 1.72 | 89.9 | 0.09 |
| Occipital | 1.51 | 90.3 | 0.1 |  |  |  | 0.42 | 71.3 | 0.7 |  |  |  |
| Insula | 1.10 | 86.6 | 0.3 |  |  |  | -0.20 | 86.6 | 0.8 |  |  |  |
| Hippocampus | 0.61 | 90.3 | 0.5 |  |  |  | 0.14 | 79.1 | 0.9 |  |  |  |
| Cingulate | 2.23 | 91.0 | **0.03** | -1.63 | 89.8 | 0.1 | -1.81 | 41.8 | 0.08 |  |  |  |
| Amygdala | -0.28 | 88.5 | 0.8 |  |  |  | 0.76 | 83.5 | 0.5 |  |  |  |
| Caudate | 0.25 | 89.2 | 0.8 |  |  |  | -0.97 | 82.4 | 0.3 |  |  |  |
| Putamen | 0.78 | 90.8 | 0.4 |  |  |  | 0.19 | 58.2 | 0.9 |  |  |  |
| Thalamus | 1.36 | 90.6 | 0.2 |  |  |  | 0.08 | 90.7 | 0.9 |  |  |  |
|  |  |  |  |  |  |  |  |  |  |  |  |  |
| **Insula cortex functional network** | | | |  |  |  |  |  |  |  |  |  |
| Whole brain | 3.67 | 88.5 | **0.0004** | -3.30 | 86.1 | **0.001** | -0.75 | 88.7 | 0.5 |  |  |  |
| Frontal | 3.00 | 85.8 | **0.004** | -2.55 | 84.6 | **0.01** | -2.07 | 84.3 | **0.04** | 1.82 | 79.3 | 0.07 |
| Temporal | 4.79 | 88.7 | **<0.00001** | -4.53 | 87.7 | **<0.00001** | -2.13 | 87.9 | **0.04** | 1.75 | 83.2 | 0.08 |
| Parietal | 3.61 | 86.2 | **0.0005** | -3.21 | 85.1 | **0.002** | -1.95 | 88.7 | 0.06 |  |  |  |
| Occipital | 1.61 | 90.4 | 0.1 |  |  |  | 0.76 | 88.4 | 0.5 |  |  |  |
| Insula | 0.55 | 86.4 | 0.6 |  |  |  | -1.16 | 88.9 | 0.3 |  |  |  |
| Hippocampus | 1.22 | 90.2 | 0.2 |  |  |  | -0.39 | 88.1 | 0.7 |  |  |  |
| Cingulate | 2.22 | 90.7 | **0.03** | -1.40 | 90.5 | 0.2 | -2.14 | 90.6 | **0.04** | 2.38 | 87.2 | **0.019** |
| Amygdala | 0.51 | 88.5 | 0.6 |  |  |  | -0.53 | 87.1 | 0.6 |  |  |  |
| Caudate | -0.08 | 89.1 | 0.9 |  |  |  | -2.55 | 89.7 | **0.01** | 2.77 | 84.2 | **0.007** |
| Putamen | 1.05 | 90.8 | 0.3 |  |  |  | 1.08 | 91.0 | 0.3 |  |  |  |
| Thalamus | 1.53 | 90.5 | 0.1 |  |  |  | 1.39 | 88.2 | 0.2 |  |  |  |
|  |  |  |  |  |  |  |  |  |  |  |  |  |
| **Cingulate functional network** | | | |  |  |  |  |  |  |  |  |  |
| Whole brain | -1.25 | 90.6 | 0.2 |  |  |  | -1.25 | 90.6 | 0.2 |  |  |  |
| Frontal | -1.89 | 87.0 | 0.06 |  |  |  | -1.89 | 87.0 | 0.06 | 1.67 | 81.2 | 0.1 |
| Temporal | -1.66 | 89.4 | 0.1 |  |  |  | -1.66 | 89.4 | 0.1 |  |  |  |
| Parietal | -2.12 | 90.0 | **0.04** | 1.36 | 84.7 | 0.18 | -2.12 | 90.0 | **0.04** |  |  |  |
| Occipital | 0.50 | 88.9 | 0.6 |  |  |  | 0.50 | 88.9 | 0.6 |  |  |  |
| Insula | 0.38 | 90.5 | 0.7 |  |  |  | 0.38 | 90.5 | 0.7 |  |  |  |
| Hippocampus | -0.13 | 88.6 | 0.9 |  |  |  | -0.13 | 88.6 | 0.9 |  |  |  |
| Cingulate | -1.94 | 81.4 | 0.06 |  |  |  | -1.94 | 81.4 | 0.06 |  |  |  |
| Amygdala | 1.07 | 88.8 | 0.3 |  |  |  | 1.07 | 88.8 | 0.3 |  |  |  |
| Caudate | -0.75 | 90.9 | 0.5 |  |  |  | -0.75 | 90.9 | 0.5 |  |  |  |
| Putamen | 1.27 | 89.6 | 0.2 |  |  |  | 1.27 | 89.6 | 0.2 |  |  |  |
| Thalamus | 0.95 | 90.0 | 0.3 |  |  |  | 0.95 | 90.0 | 0.3 |  |  |  |
|  |  |  |  |  |  |  |  |  |  |  |  |  |
| **Hippocampus functional network** | | | |  |  |  |  |  |  |  |  |  |
| Whole brain | 3.35 | 85.6 | **0.001** | -2.37 | 84.0 | **0.02** | -0.74 | 90.8 | 0.5 |  |  |  |
| Frontal | 3.74 | 89.1 | **0.0003** | -3.35 | 86.8 | **0.001** | -2.00 | 88.0 | **0.05** | 1.92 | 83.6 | 0.06 |
| Temporal | 3.23 | 86.7 | **0.002** | -2.51 | 84.6 | **0.01** | -1.76 | 87.5 | 0.08 |  |  |  |
| Parietal | 2.33 | 89.7 | **0.02** | -1.94 | 87.2 | 0.06 | -2.26 | 91.0 | **0.03** | 0.84 | 88.2 | 0.4 |
| Occipital | 2.24 | 91.0 | **0.03** | -1.15 | 90.0 | 0.3 | 1.22 | 88.9 | 0.2 |  |  |  |
| Insula | 1.11 | 87.7 | 0.3 |  |  |  | -0.09 | 91.0 | 0.9 |  |  |  |
| Hippocampus | 1.23 | 89.6 | 0.2 |  |  |  | -0.66 | 86.2 | 0.5 |  |  |  |
| Cingulate | 2.24 | 91.0 | **0.03** | -1.15 | 90.0 | 0.3 | -2.11 | 73.0 | **0.04** | 2.70 | 90.5 | **0.008** |
| Amygdala | 0.28 | 87.6 | 0.8 |  |  |  | 0.03 | 88.1 | 1.0 |  |  |  |
| Caudate | 0.10 | 89.5 | 0.9 |  |  |  | -1.09 | 90.9 | 0.3 |  |  |  |
| Putamen | 0.58 | 90.7 | 0.6 |  |  |  | 1.32 | 89.9 | 0.2 |  |  |  |
| Thalamus | 1.60 | 90.4 | 0.1 |  |  |  | 0.95 | 88.9 | 0.3 |  |  |  |
|  |  |  |  |  |  |  |  |  |  |  |  |  |
| **Hubs functional network** | | | |  |  |  |  |  |  |  |  |  |
| Whole brain | 2.91 | 86.0 | **0.005** | -2.46 | 83.7 | **0.02** | -0.68 | 89.8 | 0.5 |  |  |  |
| Frontal | 3.47 | 89.7 | **0.0008** | -3.20 | 87.1 | **0.002** | -1.74 | 89.4 | 0.09 |  |  |  |
| Temporal | 2.63 | 86.8 | **0.01** | -2.26 | 84.7 | **0.03** | -1.34 | 78.9 | 0.2 |  |  |  |
| Parietal | 1.68 | 90.5 | 0.1 |  |  |  | -2.62 | 87.9 | **0.01** | 1.08 | 89.6 | 0.3 |
| Occipital | 1.92 | 90.9 | 0.06 |  |  |  | 0.45 | 76.2 | 0.7 |  |  |  |
| Insula | 1.04 | 87.4 | 0.3 |  |  |  | 0.06 | 90.1 | 1.0 |  |  |  |
| Hippocampus | 0.43 | 90.5 | 0.7 |  |  |  | -0.24 | 85.2 | 0.8 |  |  |  |
| Cingulate | 1.92 | 90.9 | 0.06 |  |  |  | -1.94 | 52.2 | 0.06 |  |  |  |
| Amygdala | -0.40 | 89.3 | 0.7 |  |  |  | 0.52 | 85.5 | 0.6 |  |  |  |
| Caudate | 0.12 | 89.9 | 0.9 |  |  |  | -0.83 | 87.5 | 0.4 |  |  |  |
| Putamen | 0.67 | 91.0 | 0.5 |  |  |  | 0.51 | 69.4 | 0.6 |  |  |  |
| Thalamus | 1.35 | 90.5 | 0.2 |  |  |  | 0.44 | 90.3 | 0.7 |  |  |  |

Supplementary Table 1: the correlations between the network properties and brain volume loss in brain regions, where there is a significant relationship between network measures in the frontotemporal dementia group we report the difference in rate in comparison to the gene carrier group. These results demonstrate a consistent correlation between structural and functional change between the frontal, temporal and parietal lobes greater in the frontotemporal dementia group than gene carriers. Network measures in hub regions also predicted loss of volume in the whole brain, frontal and temporal lobes greater in frontotemporal dementia than gene carriers. FTD = frontotemporal dementia

Supplementary Table 2

|  | Connection strength | | | | | | Closeness centrality | | | | | |
| --- | --- | --- | --- | --- | --- | --- | --- | --- | --- | --- | --- | --- |
|  | FTD | | | FTD vs gene carriers | | | FTD | | | FTD vs gene carriers | | |
|  | t | df | p | t | df | p | t | df | p | t | df | p |
| **Frontal lobe functional network** | | |  |  |  |  |  |  |  |  |  |  |
| MMSE | -0.39 | 80.0 | 0.7 |  |  |  | -1.60 | 80.0 | 0.1 |  |  |  |
| Logical memory – immediate z score | -1.35 | 92.0 | 0.2 |  |  |  | 0.14 | 92.0 | 0.9 |  |  |  |
| Logical memory – delayed z score | -1.23 | 92.0 | 0.2 |  |  |  | 0.34 | 92.0 | 0.7 |  |  |  |
| Forward digit span | -0.92 | 92.0 | 0.4 |  |  |  | -0.97 | 92.0 | 0.3 |  |  |  |
| Backward digit span | -1.04 | 92.0 | 0.3 |  |  |  | -1.26 | 92.0 | 0.2 |  |  |  |
| Trails A | -1.88 | 91.9 | 0.06 |  |  |  | -1.26 | 88.0 | 0.2 |  |  |  |
| Trails B | -0.79 | 92.0 | 0.4 |  |  |  | 2.46 | 92.0 | **0.02** | -1.84 | 89.9 | 0.07 |
| Digit symbol | -0.09 | 91.8 | 0.9 |  |  |  | -1.93 | 89.0 | 0.06 |  |  |  |
| Boston naming | -1.31 | 89.5 | 0.2 |  |  |  | -0.23 | 88.7 | 0.8 |  |  |  |
| Category fluency | 0.02 | 90.7 | 1.0 |  |  |  | 0.66 | 90.8 | 0.5 |  |  |  |
| Letter fluency | 0.13 | 92.0 | 0.9 |  |  |  | 0.37 | 78.5 | 0.7 |  |  |  |
| Block design | 0.90 | 92.0 | 0.4 |  |  |  | 0.82 | 86.0 | 0.4 |  |  |  |
|  |  |  |  |  |  |  |  |  |  |  |  |  |
| **Temporal lobe functional network** | | |  |  |  |  |  |  |  |  |  |  |
| MMSE | -3.58 | 80.0 | **0.0006** | -3.66 | 80.0 | **0.0005** | -1.00 | 80.0 | 0.3 |  |  |  |
| Logical memory – immediate z score | 2.04 | 92.0 | **0.04** | -1.72 | 92.0 | 0.09 | 1.09 | 92.0 | 0.3 |  |  |  |
| Logical memory – delayed z score | 2.15 | 92.0 | **0.03** | -1.72 | 92.0 | 0.09 | 1.08 | 92.0 | 0.3 |  |  |  |
| Forward digit span | 0.60 | 92.0 | 0.6 |  |  |  | -0.58 | 92.0 | 0.6 |  |  |  |
| Backward digit span | 1.14 | 91.4 | 0.3 |  |  |  | -1.37 | 92.0 | 0.2 |  |  |  |
| Trails A | 4.14 | 91.9 | **0.00008** | -3.69 | 90.5 | **0.0004** | -2.28 | 76.9 | **0.03** | 1.79 | 90.2 | 0.08 |
| Trails B | 1.25 | 91.9 | 0.2 |  |  |  | 2.73 | 91.9 | **0.008** | -1.97 | 90.3 | 0.05 |
| Digit symbol | 1.55 | 90.9 | 0.1 |  |  |  | -1.70 | 88.9 | 0.09 |  |  |  |
| Boston naming | 2.14 | 89.7 | **0.04** | -1.58 | 88.2 | 0.1 | 0.24 | 88.6 | 0.8 |  |  |  |
| Category fluency | 1.70 | 87.0 | 0.9 |  |  |  | 0.95 | 90.4 | 0.3 |  |  |  |
| Letter fluency | 1.16 | 91.4 | 0.3 |  |  |  | 0.88 | 82.4 | 0.4 |  |  |  |
| Block design | 0.51 | 92.0 | 0.6 |  |  |  | 1.72 | 89.8 | 0.09 |  |  |  |
|  |  |  |  |  |  |  |  |  |  |  |  |  |
| **Parietal lobe functional network** | | | |  |  |  |  |  |  |  |  |  |
| MMSE | 0.61 | 80.0 | 0.6 |  |  |  | -1.40 | 80.0 | 0.2 |  |  |  |
| Logical memory – immediate z score | -0.73 | 92.0 | 0.5 |  |  |  | 0.76 | 92.0 | 0.5 |  |  |  |
| Logical memory – delayed z score | -0.47 | 92.0 | 0.6 |  |  |  | 0.61 | 92.0 | 0.6 |  |  |  |
| Forward digit span | -2.20 | 79.6 | **0.03** | 2.38 | 92.0 | **0.02** | -0.62 | 92.0 | 0.5 |  |  |  |
| Backward digit span | 0.02 | 85.4 | 1.0 |  |  |  | -1.48 | 92.0 | 0.1 |  |  |  |
| Trails A | 1.90 | 86.8 | 0.06 |  |  |  | -3.17 | 92.0 | **0.002** | 2.08 | 92.0 | **0.04** |
| Trails B | -0.58 | 86.1 | 0.6 |  |  |  | 2.03 | 88.5 | **0.046** | -1.83 | 90.4 | 0.07 |
| Digit symbol | 0.43 | 86.6 | 0.7 |  |  |  | -1.76 | 81.9 | 0.08 |  |  |  |
| Boston naming | -0.83 | 84.2 | 0.4 |  |  |  | 0.98 | 88.4 | 0.3 |  |  |  |
| Category fluency | 0.15 | 83.2 | 0.9 |  |  |  | 0.99 | 91.9 | 0.3 |  |  |  |
| Letter fluency | -1.12 | 92.0 | 0.3 |  |  |  | 0.51 | 70.9 | 0.6 |  |  |  |
| Block design | -1.76 | 82.5 | 0.08 |  |  |  | 1.58 | 81.7 | 0.12 |  |  |  |
|  |  |  |  |  |  |  |  |  |  |  |  |  |
| **Occipital lobe functional network** | | | |  |  |  |  |  |  |  |  |  |
| MMSE | 2.42 | 80.0 | **0.02** | -2.33 | 80.0 | **0.02** | -2.66 | 80.0 | **0.009** | 2.52 | 80.0 | **0.01** |
| Logical memory – immediate z score | 1.78 | 92.0 | 0.08 |  |  |  | 0.14 | 92.0 | 0.9 |  |  |  |
| Logical memory – delayed z score | 1.68 | 92.0 | 0.1 |  |  |  | -0.35 | 92.0 | 0.7 |  |  |  |
| Forward digit span | 0.54 | 92.0 | 0.6 |  |  |  | -1.46 | 92.0 | 0.2 |  |  |  |
| Backward digit span | 0.75 | 92.0 | 0.5 |  |  |  | -0.30 | 78.2 | 0.8 |  |  |  |
| Trails A | 4.44 | 91.5 | **0.00003** | -4.09 | 89.7 | **0.00009** | -0.60 | 64.4 | 0.6 |  |  |  |
| Trails B | 1.60 | 92.0 | 0.1 |  |  |  | 0.89 | 69.8 | 0.4 |  |  |  |
| Digit symbol | 0.83 | 90.7 | 0.4 |  |  |  | -1.42 | 68.6 | 0.2 |  |  |  |
| Boston naming | 2.61 | 89.6 | **0.01** | -2.04 | 88.0 | **0.04** | -0.49 | 77.0 | 0.6 |  |  |  |
| Category fluency | 1.96 | 86.7 | 0.05 |  |  |  | 1.13 | 92.0 | 0.3 |  |  |  |
| Letter fluency | 0.95 | 89.7 | 0.4 |  |  |  | -0.77 | 92.0 | 0.4 |  |  |  |
| Block design | 0.49 | 92.0 | 0.6 |  |  |  | 0.86 | 62.7 | 0.4 |  |  |  |
|  |  |  |  |  |  |  |  |  |  |  |  |  |
| **Cerebellum functional network** | | | |  |  |  |  |  |  |  |  |  |
| MMSE | 1.71 | 80.0 | 0.09 |  |  |  | -2.42 | 80.0 | **0.02** | 2.61 | 80.0 | **0.01** |
| Logical memory – immediate z score | 2.12 | 92.0 | **0.04** | -2.07 | 92.0 | **0.04** | 1.17 | 92.0 | 0.3 |  |  |  |
| Logical memory – delayed z score | 1.58 | 92.0 | 0.1 |  |  |  | 0.89 | 92.0 | 0.4 |  |  |  |
| Forward digit span | 0.53 | 92.0 | 0.6 |  |  |  | -0.30 | 92.0 | 0.8 |  |  |  |
| Backward digit span | 1.19 | 91.9 | 0.24 |  |  |  | 0.01 | 77.9 | 1.0 |  |  |  |
| Trails A | 2.91 | 91.0 | **0.005** | -2.34 | 89.4 | **0.02** | -2.00 | 75.6 | 0.05 |  |  |  |
| Trails B | 1.25 | 91.7 | 0.2 |  |  |  | 2.41 | 91.7 | **0.02** | -1.69 | 88.6 | 0.1 |
| Digit symbol | 0.99 | 90.4 | 0.3 |  |  |  | -1.25 | 90.1 | 0.2 |  |  |  |
| Boston naming | 1.44 | 89.4 | 0.2 |  |  |  | -0.67 | 86.2 | 0.5 |  |  |  |
| Category fluency | 1.60 | 87.0 | 0.1 |  |  |  | 0.69 | 91.1 | 0.5 |  |  |  |
| Letter fluency | 0.91 | 92.0 | 0.6 |  |  |  | 0.78 | 78.7 | 0.4 |  |  |  |
| Block design | 0.60 | 91.7 | 0.6 |  |  |  | 1.26 | 86.6 | 0.2 |  |  |  |
|  |  |  |  |  |  |  |  |  |  |  |  |  |
| **Insula cortex functional network** | | | |  |  |  |  |  |  |  |  |  |
| MMSE | 4.75 | 80.0 | **<0.00001** | -4.30 | 80.0 | **<0.00001** | -2.76 | 80.0 | **0.007** | 2.83 | 80.0 | **0.006** |
| Logical memory – immediate z score | 2.27 | 92.0 | **0.03** | -1.74 | 92.0 | 0.09 | -0.80 | 92.0 | 0.4 |  |  |  |
| Logical memory – delayed z score | 2.12 | 92.0 | **0.04** | -1.51 | 92.0 | 0.1 | -0.34 | 92.0 | 0.7 |  |  |  |
| Forward digit span | 0.70 | 92.0 | 0.5 |  |  |  | -0.79 | 92.0 | 0.4 |  |  |  |
| Backward digit span | 0.88 | 91.7 | 0.4 |  |  |  | -0.18 | 92.0 | 0.9 |  |  |  |
| Trails A | 3.41 | 91.8 | **0.001** | -2.95 | 90.1 | **0.004** | -1.63 | 92.0 | 0.1 |  |  |  |
| Trails B | 2.55 | 92.0 | **0.012** | -1.92 | 90.6 | 0.06 | 1.77 | 91.9 | 0.08 |  |  |  |
| Digit symbol | 1.30 | 90.6 | 0.2 |  |  |  | -2.24 | 92.0 | **0.03** | 1.07 | 87.9 | 0.3 |
| Boston naming | 2.29 | 89.8 | **0.02** | -1.58 | 88.7 | 0.1 | -2.15 | 88.6 | **0.03** | 1.25 | 86.0 | 0.2 |
| Category fluency | 1.80 | 84.8 | 0.08 |  |  |  | -0.24 | 89.1 | 0.8 |  |  |  |
| Letter fluency | 1.16 | 91.5 | 0.25 |  |  |  | 0.44 | 89.7 | 0.7 |  |  |  |
| Block design | 0.82 | 91.0 | 0.42 |  |  |  | -0.08 | 91.4 | 0.9 |  |  |  |
|  |  |  |  |  |  |  |  |  |  |  |  |  |
| **Cingulate functional network** | | | |  |  |  |  |  |  |  |  |  |
| MMSE | 3.09 | 80.0 | **0.003** | -2.94 | 80.0 | **0.004** | -0.99 | 80.0 | 0.3 |  |  |  |
| Logical memory – immediate z score | 2.05 | 92.0 | **0.04** |  |  |  | 0.60 | 92.0 | 0.6 |  |  |  |
| Logical memory – delayed z score | 1.82 | 92.0 | 0.07 |  |  |  | 0.26 | 92.0 | 0.8 |  |  |  |
| Forward digit span | -0.05 | 92.0 | 1.0 |  |  |  | -0.83 | 92.0 | 0.4 |  |  |  |
| Backward digit span | 0.38 | 91.5 | 0.7 |  |  |  | -0.71 | 70.7 | 0.5 |  |  |  |
| Trails A | 3.22 | 90.4 | **0.002** | -3.00 | 89.3 | **0.004** | -1.75 | 87.5 | 0.08 |  |  |  |
| Trails B | 1.74 | 91.0 | 0.09 |  |  |  | 1.86 | 91.6 | 0.07 |  |  |  |
| Digit symbol | 0.37 | 89.5 | 0.7 |  |  |  | -1.45 | 90.2 | 0.2 |  |  |  |
| Boston naming | 2.66 | 88.6 | **0.009** | -2.03 | 88.6 | **0.04** | 0.10 | 89.5 | 0.9 |  |  |  |
| Category fluency | 2.37 | 83.1 | **0.02** | -1.48 | 82.2 | 0.1 | 1.07 | 90.1 | 0.3 |  |  |  |
| Letter fluency | 1.15 | 91.5 | 0.3 |  |  |  | 0.19 | 79.5 | 0.9 |  |  |  |
| Block design | 0.22 | 90.4 | 0.8 |  |  |  | 1.05 | 87.5 | 0.3 |  |  |  |
|  |  |  |  |  |  |  |  |  |  |  |  |  |
| **Hippocampus functional network** | | | |  |  |  |  |  |  |  |  |  |
| MMSE | 3.81 | 80.0 | **0.0003** | -3.49 | 80.0 | **0.0008** | -1.52 | 80.0 | 0.1 |  |  |  |
| Logical memory – immediate z score | 2.55 | 92.0 | **0.01** | -1.91 | 92.0 | 0.06 | 0.22 | 92.0 | 0.8 |  |  |  |
| Logical memory – delayed z score | 1.99 | 92.0 | 0.05 |  |  |  | 0.84 | 92.0 | 0.4 |  |  |  |
| Forward digit span | 0.55 | 92.0 | 0.6 |  |  |  | -1.03 | 92.0 | 0.3 |  |  |  |
| Backward digit span | 0.89 | 92.0 | 0.4 |  |  |  | -1.41 | 92.0 | 0.2 |  |  |  |
| Trails A | 2.41 | 91.5 | **0.02** | -2.02 | 89.9 | **0.04** | -1.88 | 83.5 | 0.06 |  |  |  |
| Trails B | 0.44 | 91.9 | 0.7 |  |  |  | 2.31 | 91.7 | **0.02** | -2.02 | 90.0 | **0.04** |
| Digit symbol | 1.62 | 90.7 | 0.1 |  |  |  | -1.53 | 90.9 | 0.1 |  |  |  |
| Boston naming | 1.33 | 89.2 | 0.2 |  |  |  | -1.70 | 69.4 | 0.09 |  |  |  |
| Category fluency | 1.50 | 87.4 | 0.1 |  |  |  | 0.39 | 91.3 | 0.7 |  |  |  |
| Letter fluency | 0.87 | 91.9 | 0.4 |  |  |  | 0.43 | 92.0 | 0.7 |  |  |  |
| Block design | 0.84 | 91.7 | 0.4 |  |  |  | 1.67 | 88.9 | 0.1 |  |  |  |
|  |  |  |  |  |  |  |  |  |  |  |  |  |
| **Hubs functional network** | | | |  |  |  |  |  |  |  |  |  |
| MMSE | 2.55 | 80.0 | **0.01** | -2.44 | 80.0 | **0.02** | -2.13 | 80.0 | **0.04** | 2.06 | 80.0 | **0.04** |
| Logical memory – immediate z score | 1.71 | 92.0 | 0.09 |  |  |  | 0.10 | 92.0 | 0.9 |  |  |  |
| Logical memory – delayed z score | 1.58 | 92.0 | 0.1 |  |  |  | 0.02 | 92.0 | 1.0 |  |  |  |
| Forward digit span | 0.42 | 92.0 | 0.7 |  |  |  | -1.10 | 92.0 | 0.3 |  |  |  |
| Backward digit span | 0.78 | 91.7 | 0.4 |  |  |  | -0.33 | 73.2 | 0.7 |  |  |  |
| Trails A | 4.33 | 91.7 | **0.00004** | -3.89 | 89.7 | **0.0002** | -0.40 | 79.8 | 0.7 |  |  |  |
| Trails B | 1.62 | 92.0 | 0.1 |  |  |  | 2.16 | 89.1 | **0.03** | -1.32 | 90.4 | 0.2 |
| Digit symbol | 1.01 | 91.2 | 0.3 |  |  |  | -1.52 | 75.6 | 0.1 |  |  |  |
| Boston naming | 2.39 | 89.9 | **0.02** | -1.78 | 87.9 | 0.08 | 0.20 | 86.0 | 0.8 |  |  |  |
| Category fluency | 2.01 | 87.7 | **0.04** | -1.30 | 85.0 | 0.2 | 1.15 | 91.8 | 0.3 |  |  |  |
| Letter fluency | 0.91 | 88.3 | 0.4 |  |  |  | 0.08 | 62.1 | 0.9 |  |  |  |
| Block design | 0.84 | 92.0 | 0.4 |  |  |  | 1.46 | 78.6 | 0.2 |  |  |  |

Supplementary Table 2: The relationship between cognitive scores and network meausures, where there is a significant relationship between network measures in the frontotemporal dementia group we report the difference in rate in comparison to the gene carrier group. Cognitive tests where there is a significant ceiling effect (MMSE and Trails A) demonstrate a relationship with network measures, particular connection strength. FTD=frontotemporal dementia
